# Femurs in patients with hip dysplasia have fundamental shape differences compared with cam femoroacetabular impingement

**DOI:** 10.1093/jhps/hnae004

**Published:** 2024-02-05

**Authors:** Michael D Harris, Brecca M.M Gaffney, John C Clohisy, Cecilia Pascual-Garrido

**Affiliations:** Program in Physical Therapy, Department of Orthopaedic Surgery, Washington University School of Medicine, 4444 Forest Park Ave, St Louis, MO 63108, USA; Department of Mechanical Engineering, University of Colorado Denver, 1200 Larimer St North Classroom Bldg, Denver, CO 80204, USA; Department of Orthopaedic Surgery, Washington University School of Medicine, 660 S. Euclid Ave., Campus Box 8233, St. Louis, MO 63110, USA; Department of Orthopaedic Surgery, Washington University School of Medicine, 660 S. Euclid Ave., Campus Box 8233, St. Louis, MO 63110, USA

## Abstract

Femoral deformities are common in developmental dysplasia of the hip (DDH), but decisions about how to treat them are not standardized. Of interest are deformities that may be akin to cam femoroacetabular impingement (FAI). We used three-dimensional and two-dimensional measures to clarify the similarities and differences in proximal femur shape variation among female patients with DDH (*n* = 68) or cam FAI (*n* = 60). Three-dimensional measures included femoral head asphericity, as well as shape variation using statistical shape modeling and principal component analysis (PCA). Two-dimensional measures included the α-angle, head–neck offset (HNO) and the neck–shaft angle (NSA). Significant shape variations were captured in the first five PCA modes, with the greatest shared variation between groups being the length from the lesser trochanter to the femoral head and greater trochanter height. Variations unique to DDH were irregularities at different areas of the femoral head, but not at the lateral femoral head–neck junction where variation was strong in FAI. The FAI group also had unique variations in greater trochanter shape. DDH femoral heads were less spherical, as indicated by larger sphere-fitting errors (*P* < 0.001). Radiographically, the DDH group had significantly smaller α-angles (*P* < 0.001), larger head–neck offsets (*P* = 0.02) and larger NSAs (*P* < 0.001). Both the articular and extra-articular regions of the proximal femur have distinct shape features in DDH and cam FAI that can uniquely affect the biomechanics of each disorder. Accordingly, approaches to addressing each disorder should be unique.

## INTRODUCTION

Developmental dysplasia of the hip (DDH) is a known etiological factor in osteoarthritis and is most commonly attributed to abnormal acetabular shape [1, 2]. Periacetabular osteotomy (PAO), the most common surgical treatment for DDH, addresses the acetabular deformity by reorienting the acetabulum to better cover and stabilize the femoral head [3, 4]. However, femoral deformities are common in DDH [5–7] and there is currently no standardization about when and how to treat them.

Of particular interest when treating DDH are femoral head and neck deformities that may be akin to cam femoroacetabular impingement (cam FAI) [8–10]. Cam FAI has been well-described and, like DDH, is a known risk factor for early hip osteoarthritis [11, 12]. Reorienting the acetabulum with PAO may stabilize the dysplastic hip, but can cause secondary FAI that prolongs pain and potentiates further intra-articular joint damage [13, 14]. As a preventative measure, some surgeons perform femoral osteochondroplasty with PAO [15, 16]. Questions about when and if this adjacent procedure is warranted remain, given that unnecessary osteochondroplasty may have certain disadvantages (adhesions, over-resection, neck fracture) [17].

Because patients with DDH and cam FAI may present with similar symptoms [18, 19], it is likely that the same physicians will treat both groups of patients. Previous research has described the radiographic characteristics of femur shape in DDH or cam FAI cases separately [20–23], but a more direct comparison between groups may help inform treatment approaches that can be shared or must be unique for each group. Also, while two-dimensional (2D) descriptions of femoral shape are most common and are readily obtained in clinic, three-dimensional (3D) measures more completely describe shape variations across the entire head and neck that can affect joint loading. Like previous 2D analyses, 3D analyses of femurs, such as analyses using statistical shape modeling (SSM), have been performed separately on DDH and cam FAI populations, but rarely with a direct comparison [24, 25]. Thus, the objective of this study was to directly compare 3D and 2D proximal femoral shape and shape variation among patients with DDH and patients with cam FAI who were similar in sex, age and clinical presentation to clarify their similarities and differences.

## MATERIALS AND METHODS

With Institutional Review Board approval, computed tomography (CT) images, plain films and clinical history were retrospectively collected from female patients with a primary diagnosis of either DDH or cam FAI and scheduled for surgery between January 2008 and September 2018. Because females represent 72–84% of symptomatic patients with DDH [26], only female patients were selected to eliminate geometric differences related to sex [27]. Inclusion criteria for patients with DDH were a lateral center edge angle (LCEA) <20° and hip or groin pain lasting greater than 3 months. No criterion for alpha angle, the key measure for cam FAI head-neck deformity, was specified for the DDH group. Inclusion criteria for patients with cam FAI included a Dunn-view alpha angle >55°, a LCEA >20° and hip or groin pain lasting greater than 3 months. All radiographs were collected using a standardized protocol including standing anteroposterior pelvis and supine Dunn lateral views. Exclusion criteria for both groups were a BMI >30 kg/m^2^; history of Legg-Calves-Perthes disease, avascular necrosis or slipped capital femoral epiphysis; advanced osteoarthritis (Tonnis Grade III–IV); no CT prior to surgery; and documented prior ipsilateral PAO or femoral osteochondroplasty. Of 359 potential candidates, 68 patients with DDH (27.6 ± 8.3 y/o, 24.5 ± 2.9 kg/m^2^ BMI) and 60 patients with cam FAI (29.9 ± 12.2 y/o, 22.7 ± 2.8 kg/m^2^ BMI) were included in the study.

### 3D measures: femoral head sphericity & proximal femur statistical shape modeling

**Fig. 1.**
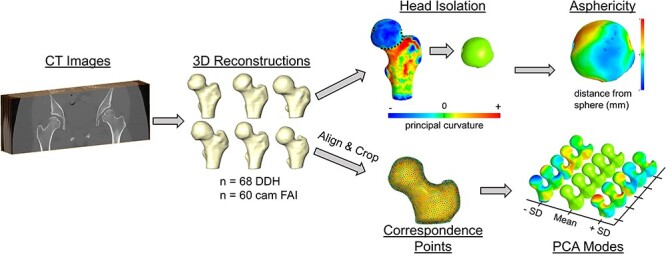
3D descriptors of femoral head asphericity and proximal femur shape variation. Asphericity—subject-specific 3D reconstructions from CT were cut at the head–neck junction using principal curvature, and spheres were fit to each head; the distance between the head and sphere was calculated across the entire head surface and fitting errors were determined. Shape Variation—3D reconstructions were tightly aligned and cropped at the lesser trochanter; correspondence points were then placed on each femur and SSM was applied to determine the proximal femur variation that existed in DDH and FAI groups; shape variation was presented using PCA modes.

3D reconstructions of the proximal femurs on the symptomatic limb were generated from the CT images using Amira software (v2022.1; Thermo Fisher). All CT images were collected as part of standard-of-care, using a low-dose protocol from the anterior superior iliac spine of the pelvis to the lesser trochanter of the femur (voxel resolution 0.8 × 0.8 × 0.6 mm) [28]. The femoral head was objectively isolated at the head–neck junction and fit to a sphere using FEBio software and established methods [29] (Fig. 1). Asphericity was quantified with a ‘fitting error’ based on the root-mean-squared distance between nodes on the native articular geometry and the best-fit sphere; lower fitting errors indicated better fit [29].

For SSM, the proximal femur reconstructions from all subjects were tightly aligned using an iterative closest point alignment algorithm [25]. Uniform scaling was allowed during alignment to reduce shape variation caused by overall size differences among the femurs and to lower root mean square alignment errors. Next, each femur was cropped at the medial-most aspect of the lesser trochanter (Fig. 1).

SSM was then performed with an established iterative correspondence particle positioning strategy using ShapeWorks software (Fig. 1) [30]. Using 4096 correspondence points, the mean femoral shapes for the DDH and FAI groups were derived. Principal component analysis (PCA) was then used to describe the primary modes of shape variation in each group. The PCA modes utilize eigenvectors and eigenvalues of the correspondence point covariance matrix to rank the direction and amount of shape variation, with PCA mode 1 describing the greatest amount of variation.

### 2D measures: radiographic descriptors

**Fig. 2.**
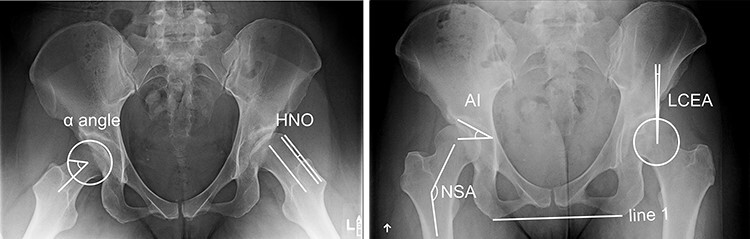
Radiographic descriptors of the femoral head and acetabulum. The α angle was measured as the angle between the femoral neck axis and the point of beginning asphericity. HNO was the distance between a line parallel to the femoral neck axis and tangent to the anterolateral neck, and a second parallel line tangent to the anterolateral head. NSA on the anteroposterior view was the angle between the femoral neck axis and the long axis of the femur. AI was the angle between a line parallel to the inter-ischial-tuberosity line (line 1) at the inferior aspect of the acetabular sourcil and a second line from the inferior to lateral aspects of the sourcil. LCEA was the angle between a line perpendicular to line 1 originating from the center of a femoral head best-fit circle and a second line from the center of the circle to the lateral aspect of acetabular sourcil.

Radiographic descriptors of femoral head shape were measured with the alpha angle and HNO in the Dunn lateral view, and the NSA in the anteroposterior view of plain films (Fig. 2) [31–33]. Additionally, acetabular geometry was measured using acetabular inclination (AI) and the LCEA (Fig. 2) [34, 35]. Inter-observer and intra-observer reliability of the radiographic analyses was previously performed [36].

### Statistical analysis

Parallel analysis was used to determine the PCA modes containing significant shape variation. Variance accounted for in the significant modes was calculated and the physical meaning of shape variation differences between the DDH and FAI groups was described at two standard deviations from the mean in each mode. Inter-group differences in radiographic measures and fitting errors were tested using Welch’s robust *t*-test (α = 0.05) to account for unequal sample sizes and potential inhomogeneity of variance.

## RESULTS

### 3D measures

Femoral heads in the DDH group were significantly less spherical than the cam FAI group, as indicated by larger fitting errors (0.66 ± 0.17 mm versus 0.53 ± 0.15 mm, *P* < 0.001).

The first five PCA modes described significant shape variation in both the DDH and cam FAI groups and collectively accounted for 86.9% of the total variation in DDH and 88.0% in FAI (Suppl. Fig 1).

**Fig. 3.**
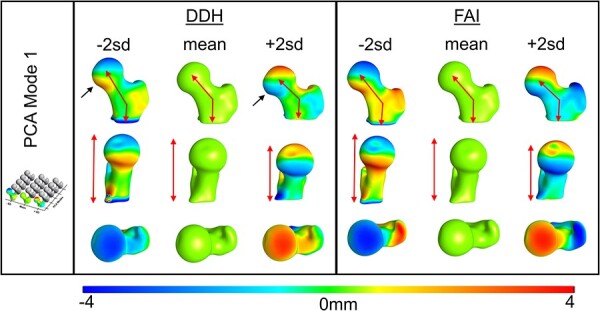
PCA Mode 1 contained 53.76% and 41.63% of the total DDH and FAI variation, respectively. Color plots show the distance from the mean at ± 2 SD. Common shape variation (red arrows) was in the NSA and associated vertical distance from the lesser trochanter to the superior femoral head. DDH femurs had unique variation (black arrows) in the shape of the femoral head.

**Fig. 4.**
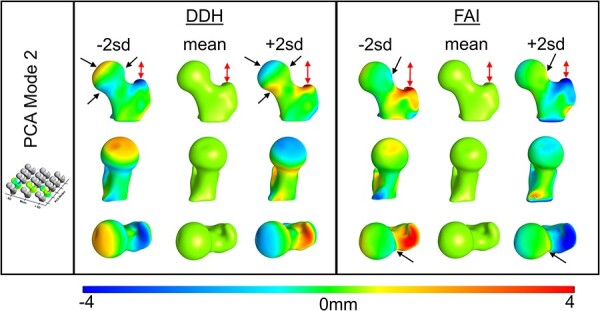
PCA Mode 2 contained 13.10% and 20.96% of the total DDH and FAI variation, respectively. Color plots show the distance from the mean at ± 2 SD. Common shape variation (red arrows) was in the height of the greater trochanter relative to the femoral head, with greater variation seen in the FAI group. DDH femurs had unique variation (black arrows) in the shape of the femoral head. FAI femurs had unique variation in head–neck junction shape.

**Fig. 5.**
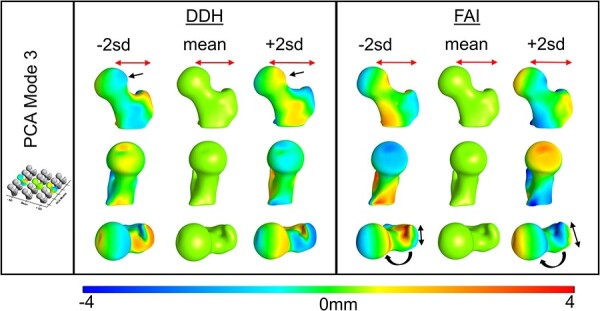
PCA Mode 3 contained 9.11% and 12.86% of the total DDH and FAI variation, respectively. Color plots show the distance from the mean at ± 2 SD. Common shape variation (red arrows) was in the horizontal femoral offset. DDH femurs had unique variation (black arrows) in the shape of the femoral head. FAI femurs had unique variation in the contour of the great trochanter and proximal femoral torsion.

Within the PCA modes, the DDH and FAI groups had shared 3D shape variation as well as variation that was unique to each group. The greatest shared shape variation for both groups (i.e. PCA Mode 1) was in the vertical height from the lesser trochanter to the femoral head (Fig. 3), which included high variation in the femoral NSA (Suppl Video 1). Unique to DDH, PCA Mode 1 revealed irregular variation in the femoral head (Fig. 3). In Mode 2, both groups showed variation in greater trochanter height with the DDH group again revealing unique shape irregularity at different areas of the femoral head, and the FAI group demonstrating variation in the lateral head–neck junction (Fig. 4, Suppl Video 2). In Mode 3, both groups had femoral offset variation, with unique variation occurring in femoral head shape of DDH, and the greater trochanter contour and femoral version of the FAI group (Fig. 5, Suppl Video 3). Modes 4 and 5 described more subtle shape variation. Shared variation in Mode 4 was primarily in the anterior–posterior width of the femur, with unique variation in greater trochanter curvature of the FAI group (Suppl Fig. 2, Suppl Video 4). Mode 5 had no notable shared variation, but did capture unique variation in the head–neck shape of the FAI group. Mode 5 also captured femoral torsion variation in the DDH group (Suppl Fig. 3, Suppl Video 5).

### 2D measures

The femurs in the DDH group had significantly smaller alpha angles than the cam FAI group (56.2° ± 17.1° versus 64.9° ± 7.7°, *P* < 0.001), significantly larger HNO (5.4 ± 1.9 mm versus 4.6 ± 1.6 mm, *P* = 0.02) and significantly larger NSAs (140.6° ± 7.5° versus 135.7° ± 5.1°, *P* < 0.001). On the acetabular side, LCEAs were significantly smaller in the DDH group (13.6° ± 7.0° versus 29.0° ± 6.9°, *P* < 0.001), and AI values were significantly larger (15.8° ± 5.5° versus 4.4° ± 6.9°, *P* < 0.001).

## DISCUSSION

The objective of this study was to clarify the similarities and differences in proximal femoral shape and shape variation among patients with DDH and patients with cam FAI. Unique 3D shape variations were found, including irregularities in various areas of the head among patients with DDH and more variation in the greater trochanter in FAI. Significant differences were also evident in the radiographic measures and 3D asphericity, with femoral heads in patients with DDH being significantly less spherical than those with cam FAI. The two groups did share some shape variations related to vertical height from the lesser trochanter to the femoral head, greater trochanter height and femoral offset. Mechanistically, shape abnormalities of the femur affect joint loading and influence cartilage wear patterns [37–40], which can be different between groups. For physicians treating female patients with DDH or cam FAI, it can be important to know the distinct inter-group shape differences and that they occur both at the intra- and extra-articular regions of the proximal femur.

The shape features unique to each group provide important distinctions between DDH and cam FAI populations. Patients in the DDH group had greater 3D head shape variation as demonstrated by irregularities of the femoral head captured by the first three major PCA modes. While the irregularities within the cam FAI group were most noticeable in the anterolateral head neck junction, irregularities in the DDH heads occurred in various regions of the femoral head. Consistent with previous reports, both groups of patients had radiographically abnormal femur shape [5, 6, 21], but again, the groups had important differences. First, patients with DDH had smaller average alpha angles and larger head–neck offsets. However, the average alpha angle in DDH was 56°, which is near the commonly used cutoff of 55° to distinguish a head shape deformity [41, 42]. It is important to note that despite using the Dunn lateral view, this alpha angle variation in patients with DDH was not consistently in the anterolateral head neck region of the femoral head and may not necessarily signify cam lesions. Femoral heads in DDH were significantly less spherical than those in cam FAI, and alpha angle variation was greater (standard deviation = 17° versus 7° in cam FAI). Also, while head–neck offsets in patients with DDH were larger than cam FAI, both groups had average head–neck offsets smaller than published values for healthy hips (normal = 7–9+ mm) [29, 31]. Additionally, femurs in the DDH group had larger NSAs than those in the cam FAI group, and would typically be considered coxa valga, while NSAs in the cam FAI group fell within normal ranges for females [43].

These findings emphasize the challenge when deciding on clinical treatment in DDH cases. For example, the presence of cam lesions or abnormal HNO leads to more severe cartilage wear in the acetabulum [37, 38]. However, that relationship between geometry and damage is not universally true, and many hips shown to have radiographic evidence of cam lesions are not symptomatic [44, 45]. Prior reports indicate that if only the cam lesion in DDH cases is treated, patients’ outcomes are more likely to be negative [46]. Yet, outcomes of correcting femoral coverage with PAO, combined with femoral osteochondroplasty, are mixed compared with PAO alone [15, 47]. Our data support that there are some female patients with DDH with classical cam-type lesions and reduced HNO [8, 48]. The average and range of shape variations of these lesions were less pronounced than in the patients without DDH. Femoral head–neck osteochondroplasty might be appropriate in such cases for reducing the possibility of secondary FAI after PAO. Surgeons should carefully consider the location and morphology of the deformity in context with the shape of the acetabulum to estimate if secondary FAI is, in fact, likely after acetabular rotation. Checking range of motion following the PAO correction is critical for deciding if osteochondroplasty should be performed. For many other patients with DDH and irregular heads (e.g. oblong heads) and no conclusive impingement, traditional osteochondroplasty in the anterior head–neck area may be an unnecessary procedure that could damage the integrity of the femoral head and neck. Our results support that presence of femoral head deformity, even with a higher-than-normal alpha angle, should not automatically indicate osteochondroplasty. Future studies can couple the shape models established here with high-accuracy kinematics to identify femoral shape features that best indicate when osteochondroplasty is needed.

The relationship between proximal femur shape and symptom manifestation can be complex. Interestingly, prior studies suggest that most patients with DDH have a positive hip impingement sign (FADIR test) on clinical exam [49] despite radiographic cam being found in the minority of cases [8, 48]. This common clinical finding might be related to rim disease rather than true impingement in the anterolateral head–neck area. In context with those prior studies, the shape findings in the current study lead to the question, is impingement the same in DDH and cam FAI or do they differ mechanically while causing a similar clinical response? Conversely, some patients with DDH and abnormal femur shape may not manifest symptoms of impingement due to severely shallow acetabular shape or excessive femoral anteversion that can functionally ‘protect’ the hip from hip impingement.

We also found novel extra-articular variation that is important for hip joint biomechanics. First, greater trochanter curvature was more variable among femurs with cam FAI than DDH. A previous SSM study of cam FAI and healthy controls found greater trochanter curvature and height to be highly variable in both groups [25], which suggests that the lack of variation is a feature of DDH. Second, greater trochanter height variation was more prominent in cam FAI than in DDH. Third, the neck–shaft variation and proximal femur torsion was greater in DDH than in cam FAI, and has previously been shown to be more variable in DDH than in controls [24]. These differences in extra-articular femoral features are important, given the attachments of major hip muscles at the greater trochanter. Variations in greater trochanter height, curvature and distance from the hip joint center directly influence muscle moment arms and the ability of muscles to generate torque that moves or stabilizes the joint. Also, muscles are major contributors to the magnitude and orientation of contact forces inside the hip and their influence on articular mal-loading is an active area of research in both cam FAI and DDH [50, 51]. Thus, the characteristics of anatomical variation provided in the current study can be used to guide future investigations of hip joint biomechanics.

Femurs in both groups did share some shape variations. First, both groups had major variation in femoral neck extension from the lesser trochanter to the femoral head. This variation may be common among most hips, as supported by similar findings in prior SSM studies of healthy controls, patients with DDH and patients with slipped capital femoral epiphysis or Legg-Calves Perthes disease [24, 52]. There was also some shared variation in greater trochanter height and femoral width (similar to femoral offset) in both groups, which matches prior SSM reports [24, 25, 52], and may be characteristic of most femurs.

There are limitations to this study that should be considered. First, an asymptomatic control group was not included. The objective, rather, was to directly compare two demographically similar symptomatic groups who are likely to present within the same orthopedic clinics. Radiographic descriptions of proximal femurs in healthy controls are available and have been used to establish normative values [43, 53]. SSM has been previously applied to full femurs in female patients with DDH and proximal femurs in mixed-sex groups with cam FAI compared with healthy controls [24, 25]. Results from those prior studies can serve as references when interpreting results of the current study. Second, we did not include patients with borderline DDH. We found it sensible to begin with clearly defined DDH and cam FAI groups, but believe future work should involve borderline DDH cases for which there are many questions related to optimal treatments. Third, only females were included in the current study, which excludes a substantial portion of patients with symptomatic FAI. Males have been found to have larger alpha angles and it is likely that cam lesions in the current cam FAI group were smaller than would be found in a mixed-sex sample [20, 54]. This may explain why shape variation in the head–neck junction was less prominent in the current study than a prior SSM study of mixed-sex cam FAI samples [25]. However, by comparing only females with DDH or cam FAI, this study presented shape variation differences that are more related to pathology than to sex-related morphology. Finally, it is difficult to identify when the deformities observed in our cohorts developed. Patients in this study were screened for obvious radiographic signs of avascular necrosis and slipped-capital femoral epiphysis as well as notes of such in clinical records. However, patients may have received treatment in childhood, and the femoral head irregularities found could be related to type II avascular necrosis rather than solely from natural deformity development.

From this study several conclusions can be made about proximal femurs in patients with DDH or cam FAI. First, the shape of the proximal femur has unique inter-group differences evident in radiographic measurements and SSM results. Those differences occur in both the intra- and extra-articular regions of the hip and can help distinguish femur appearance and disease mechanisms within DDH versus cam FAI. Second, femoral heads in patients with DDH are less spherical than in patients with cam FAI and their asphericity is evident as irregularities that occur in several areas of the femoral head. Yet, there are commonalities between the groups, such as high variation in the femoral neck extension and femoral offset. While questions remain about the optimal treatment of femurs in DDH cases, it is important to clarify that proximal femoral deformities in DDH are unique compared with cam FAI and the approach to addressing each disease should likewise be unique.

## Supplementary Material

hnae004_Supp

## DATA AVAIILABILITY

De-identified aggregated data will be provided on reasonable request to the corresponding author.
