# Supplementary figures and images for: Femurs in patients with hip dysplasia have fundamental shape differences compared with cam femoroacetabular impingement
Source: J Hip Preserv Surg. 2024 Feb 5;11(2):132–9. doi: 10.1093/jhps/hnae004 (PMC11272640; doi:10.1093/jhps/hnae004)

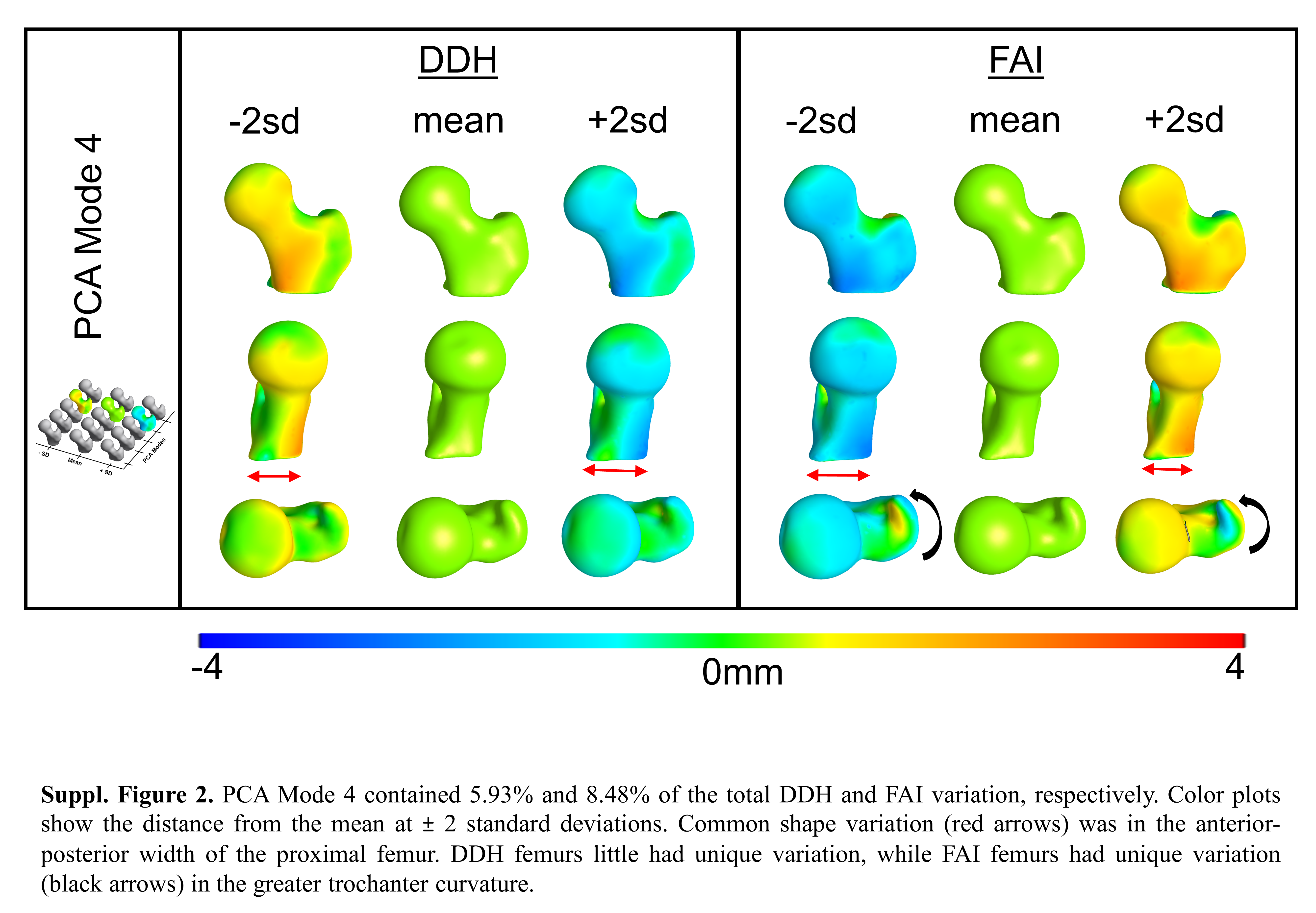

Supplement: hnae004_Supp [file hnae004_supp.zip › suppl_data/DDHvsFAI_FigureS2_wlegend.tif]

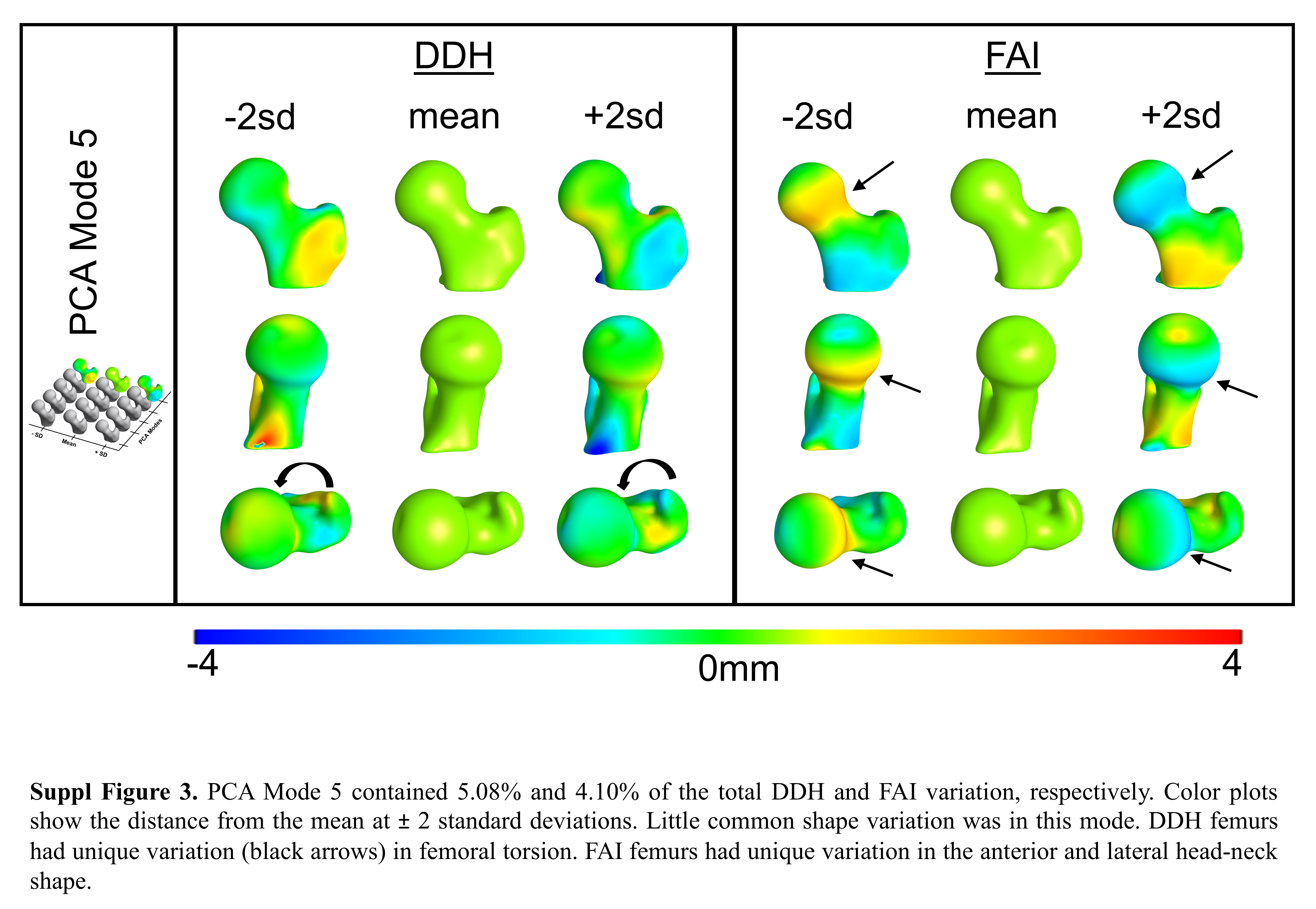

Supplement: hnae004_Supp [file hnae004_supp.zip › suppl_data/DDHvsFAI_FigureS3_wlegend.tif]
